# Metric Potential of a 3D Measurement System Based on Digital Compact Cameras

**DOI:** 10.3390/s90604178

**Published:** 2009-06-03

**Authors:** Enoc Sanz-Ablanedo, José Ramón Rodríguez-Pérez, Pedro Arias-Sánchez, Julia Armesto

**Affiliations:** 1 Geomatics Engineering Research Group. University of León, Avda. Astorga s/n, 24400 Ponferrada, Spain; E-Mail: jr.rodriguez@unileon.es; 2 Department of Natural Resources and Environmental Engineering. University of Vigo, Campus Universitario As Lagoas-Marcosende s/n, 36200 Vigo, Spain; E-Mails: parias@uvigo.es; julia@uvigo.es

**Keywords:** digital camera, photogrammetry, 3D measurement

## Abstract

This paper presents an optical measuring system based on low cost, high resolution digital cameras. Once the cameras are synchronised, the portable and adjustable system can be used to observe living beings, bodies in motion, or deformations of very different sizes. Each of the cameras has been modelled individually and studied with regard to the photogrammetric potential of the system. We have investigated the photogrammetric precision obtained from the crossing of rays, the repeatability of results, and the accuracy of the coordinates obtained. Systematic and random errors are identified in validity assessment of the definition of the precision of the system from crossing of rays or from marking residuals in images. The results have clearly demonstrated the capability of a low-cost multiple-camera system to measure with sub-millimetre precision.

## 1. Introduction

Close-range photogrammetry is a technique used to obtain the 3D coordinates of an object from two or more photographs of it. In analog photography, photogrammetric cameras have special features such as fiducial marks or systems to ensure the flatness of the film. The replacement of film with digital sensors removes the need for such elements because the array of pixels can be used to set a camera coordinate system. Although any digital camera can become a measuring instrument, it is agreed that the term “metric camera” should be reserved for those cameras specifically designed for photogrammetric tasks, such as the Rollei D7 Metric [1]. These cameras have a robust mechanical structure, using well-aligned lenses with low distortion and lack an autofocus or other technologies that can uncontrollably change the internal geometry of the camera. In keeping with this approach, all other cameras should be referred to as non-metric cameras, regardless of whether they may share some features such as a robust sensor body or high quality lenses. In non-metric cameras, technologies such as autofocus, zoom lenses, retrofocus constructions, image stabilisers, among others, are far from being useful to photogrammetrists and can in fact reduce the potential accuracy of a given camera [2]. In these cameras, we can distinguish two categories from the standpoint of their metric potential: professional (or high-grade, high quality, etc.) grade cameras and consumer (or amateur, low cost, etc.) grade cameras. Professional cameras have features such as a robust structure, a large sensor with high resolution and sensitivity, good lens quality, the ability to exchange lenses, while consumer-grade cameras include models that may include any of these features all the way down to compact cameras, as used in this work, where the lens is packaged together inside the camera.

The most important difference in consumer cameras as compared to professional cameras is their lower geometric stability. This issue involves lower reliability and durability over time of the modellization of the internal geometry of the cameras. In response to this problem algorithms and rapid calibration procedures [3] have been developed in recent years that allow these cameras to be used for photogrammetric applications [4,5].

Some examples of photogrammetric work performed with consumer grade cameras are given in [6] in which Ricoh 6000 and Kodak DX 3500 cameras are used in documentation of agro-industrial heritage, in [7], where the Olympus C-5050 is used in architectural surveys, in [8] in which a Sony DSC-F707 is used to measure the deflection of a loaded beam, [9], which uses the Olympus E-20 compact camera to measure wrinkling of a gossamer spacecraft membrane and obtains an accuracy of 1/80,000, [10], in which a Kodak DC290 is used to perform precision measurements in space structures, [11], which examines the geometric stability of the Nikon Coolpix 5400 camera, [12], which examines the geometric stability of four consumer cameras, and [13], in which the Kodak DCS 460 professional camera is compared with the consumer grade Sony DSC-P10, Olympus C3030, and Nikon Coolpix 3100, with the result that the best accuracies are obtained with the Sony camera.

Apart from the use of digital compact cameras, another important aspect in the 3D measurement system presented here is the simultaneous use of four synchronised units. In the scientific literature, there are other optical measuring systems that use synchronised images. Some examples are [14], in which three CCD camcorders (780 by 582 pixels) are used to measure the deformation of a flexible pipe under a load, [15] in which two CCD camcorders (1,300 by 1,030 pixels) are used to control the shape of a metal beam during cooling to room temperature, [16] in which two CCD camcorders (768 by 574 pixels) are used to monitor the rupture of a concrete beam, and [17] in which two CCD camcorders (720 by 492 pixels) are used to measure the shape of a parachute during air drop tests. Unlike that proposed in this work, the systems mentioned above are generally based on camcorders, which provide less benefit in terms of resolution and dynamic range, in addition to a higher cost in terms of equipment and subsequent data processing.

## 2. Modelling and Calibration of Cameras

The purpose of modelling the cameras in the context of photogrammetric metrology is to obtain a theoretical model that describes how a scene is transformed into an image [18]. As a result of modelling, the real camera is idealised or simplified in order to express its behaviour using mathematical expressions which ultimately enable its metric uses. The performance of the measurement system depends largely on the accuracy of the modelling of the cameras.

A camera can be modelled as a spatial system that consists of a planar imaging area (electronic sensor) and a lens with a perspective centre [19]. The parameters of the interior orientation of a camera define the spatial position of the perspective centre, the principal distance, and the location of the principal point. They also encompass deviations from the principle of central perspective to include radial and tangential distortion and often image affinity and orthogonality.

**Figure 1.**
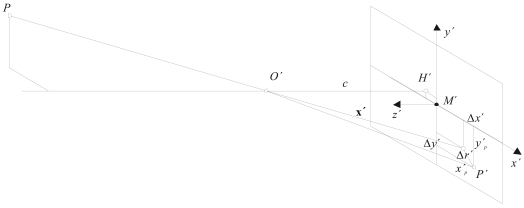
Interior orientation [19].

Figure 1 illustrates the schematic imaging process of a photogrammetric camera. Position and distance of the perspective centre and deviations from the central perspective model are described with respect to the image coordinate system as defined by means of the pixel array. The origin of the image coordinate system is located in the image plane and coincides with the perspective centre. Hence, *H*′ is the principal point, the nadir of the perspective centre *O*′ with image coordinates (*x*′_0_, *y*′_0_) approximately equal to the centre of the image *M*′. Principal distance *c* is the normal distance to the perspective centre from the image plane, approximately equal to the focal length *f* when focused at infinity. Parameters of functions describing imaging errors are dominated by the effect of radial-symmetric distortion Δ*r*′ [19].

If these parameters are known, the (error-free) imaging vector **x**′ can be defined with respect to the perspective centre (hence, the principal point):

(1) $${\text{x}}^{\prime }=\left[\begin{array}{c} x^{\prime }\\ y^{\prime }\\ z^{\prime } \end{array}\right]=\left[\begin{array}{ccc} {x^{\prime }}_{p} & -{x^{\prime }}_{0} & -\Delta x^{\prime }\\ {y^{\prime }}_{p} & -{y^{\prime }}_{0} & -\Delta y^{\prime }\\ & -c & \end{array}\right]$$

where *x*′*_p_, y*′*_p_* are the measured coordinates of image point *P*′, *x*′_0_, *y*′_0_ are the coordinates of the principal point *H*′, and Δx′, Δy′ are the axis-related correction values for image errors.

Deviations from the ideal central perspective model, attributable to image errors, are expressed in the form correction functions Δx′, Δy′ with respect to the measured image coordinates. In the first instance, measured image coordinates *x*′*_p_, y*′*_p_* are corrected by a shift of the principal point *x*′_0_, *y*′_0_:

(2) $$\begin{array}{l} x{}^{\circ}={x^{\prime }}_{p}-{x^{\prime }}_{0}\\ y{}^{\circ}={y^{\prime }}_{p}-{y^{\prime }}_{0} \end{array}$$

Hence, the image coordinates *x*°, *y*° are corrected by x′ = x° − Δx′ and *y*′ = *y*° − Δ*y*′. Strictly speaking, the values *x*°, *y*° are only approximations since the corrections Δ*x*′, Δ*y*′ must be calculated using the final image coordinates *x*′, *y*′. Consequently, correction values must be applied iteratively.

Radial (symmetric) distortion constitutes the major imaging error for most camera systems and is attributable to variations in refraction in the lens system. The radial distortion is usually modelled with a polynomial series with distortion parameters *K*_1_ and *K_n_* [20]:

(3) $$\Delta {r^{\prime }}_{\text{rad}}=K_{1}{r^{\prime }}^{3}+K_{2}{r^{\prime }}^{5}+K_{3}{r^{\prime }}^{7}+\ldots$$

where $r^{\prime }=\sqrt{x{{}^{\circ}}^{2}+y{{}^{\circ}}^{2}}$ is the image radius or distance from the principal point. The software used in this work (Photomodeler 6.0) uses the following variation:

(4) $$\Delta {r^{\prime }}_{\text{rad}}=r\left(k_{1}{r^{\prime }}^{2}+k_{2}{r^{\prime }}^{4}+k_{3}{r^{\prime }}^{6}\ldots \right)$$

Then, the image coordinates are corrected proportionally:

(5) $$\Delta {x^{\prime }}_{\text{rad}}=x^{\prime }\frac{\Delta {r^{\prime }}_{\text{rad}}}{r^{\prime }}\;\Delta {y^{\prime }}_{\text{rad}}={y^{\prime }}_{\text{rad}}\frac{\Delta {r^{\prime }}_{\text{rad}}}{r^{\prime }}$$

Radial-asymmetric distortion, often called tangential or decentering distortion, is mainly caused by decentering and misalignment of the lens and can be compensated by the following function [20]:

(6) $$\begin{array}{l} \Delta {x^{\prime }}_{\text{tan}}=B_{1}\left({r^{\prime }}^{2}+2{x^{\prime }}^{2}\right)+2B_{2}x^{\prime }y^{\prime }\\ \Delta {y^{\prime }}_{\text{tan}}=B_{2}\left({r^{\prime }}^{2}+2{y^{\prime }}^{2}\right)+2B_{1}x^{\prime }y^{\prime } \end{array}$$

Affinity and shear are used to describe deviations of the image coordinate system with respect to orthogonality and uniform scale of the coordinate axes, and can be compensated by the following function:

(7) $$\begin{array}{l} \Delta {x^{\prime }}_{\text{aff}}=C_{1}x^{\prime }+C_{2}y^{\prime }\\ \Delta {y^{\prime }}_{\text{aff}}=0 \end{array}$$

The individual terms used for modelling the imaging errors of most typical photogrammetric imaging systems can be summarised as follows:

(8) $$\begin{array}{l} \Delta x^{\prime }=\Delta {x^{\prime }}_{\text{rad}}+\Delta {x^{\prime }}_{\text{tan}}+\Delta {x^{\prime }}_{\text{aff}}\\ \Delta y^{\prime }=\Delta {y^{\prime }}_{\text{rad}}+\Delta {y^{\prime }}_{\text{tan}}+\Delta {y^{\prime }}_{\text{aff}} \end{array}$$

The procedure by which a camera is modelled is called calibration. During calibration, a system of equations is obtained that can include the parameters of interior orientation of a camera as unknowns, including parameters of functions describing imaging errors. The system of equations is then solved by minimizing errors via a procedure called bundle adjustment.

## 3. Description of the Measurement System

### 3.1. Components

The measurement system presented in this paper (Photograph 1) consists of an adjustable structure mounted on a tripod. The cameras are attached to extendable arms (40-70 cm) through a ball mount to improve the pitch. The angle of the arms with the horizontal is adjustable from 10° and 90°. Finally, the arms can rotate freely around the support shaft. This flexible adjustment of the photogrammetric network allows the permissible size of objects in optimum conditions of convergence to range from a few centimeters to 2 m.

Four Pentax Optio A40 cameras were used to take pictures. Among the most outstanding characteristics of this compact camera model (from a photogrammetric point of view), is the possibility of using a single wireless remote shutter for all of the cameras, manual control of aperture and exposure time, manual control of focus, and memory storage for position of the zoom and focus when the camera is turned off. The technical specifications of these cameras are given in Table 2.

**Figure 2.**
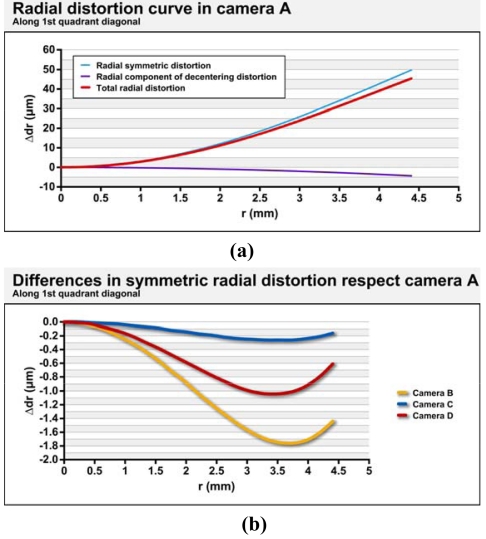

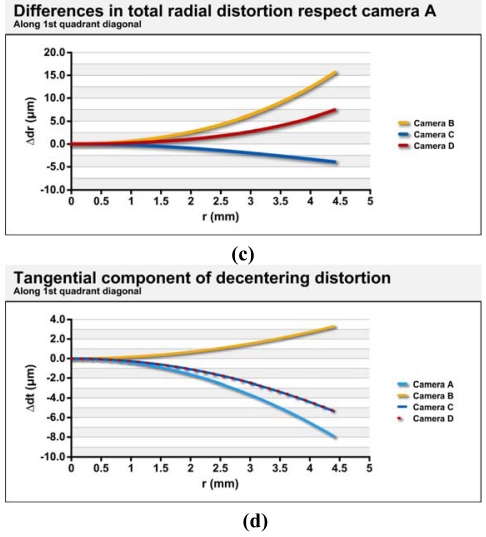
(a) Radial distortion curve in camera A, (b) differences in symmetric radial distortion curves of cameras B,C and D respect camera A, (c) differences in total radial distortion curves of cameras B,C and D respect camera A and (d) tangential component of decentering distortion curve of cameras A,B,C and D.

The cameras used as measuring equipment were independently modelled by field calibration using a plane point field (Photograph 2) and 16 convergent images from four camera stations. At each station, the camera was rotated around the optical axis by 0°, 90°, 180°, and 270°. The parameters and the quality variables for modelling the internal geometry of the cameras are shown in Table 3. Figure 2 provides distortion curves for each camera. Zero value to the third radial distortion parameter of the polynomial series was imposed because uncertainty had same magnitude as the value. Furthermore the level of correlation with the second term was over 95%. It also imposed the restriction *C*_2_ = 0 considering the pixels matrix is perfectly orthogonal.

To test the metric potential of the system, 121 white circular targets 6 mm in diameter on a black background were arranged on a flat area, (Photograph 3). Targets were distributed uniformly in a square field test (750 mm by 750 mm), with a maximum distance between targets of 1,061 mm. The targets had two concentric rings whose discontinuities represent a coding system that allows automatic referencing of homologous points. Subpixel detection algorithms were used for detection of the targets in the images.

**Figure 3.**
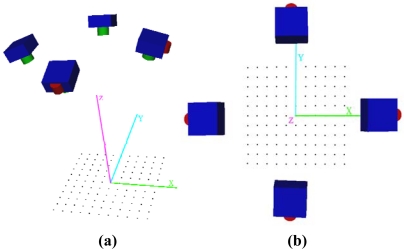
(a) Oblique view and (b) plane view of the cameras with respect to the field test.

The photogrammetric network configuration used during the tests is shown in Figure 3 and Table 4. The origin of the coordinate system was established at the target *i*_6,6_, located at the centre of the field test, with coordinates (X_c_=1,000, Y_c_=1000, Z_c_=0). The X-axis was established with the targets *i*_6,2_ and *i*_6,10_ located in the central row. The Y-axis was established at the targets *i*_10,6_ and *i*_2,6_ located in the centre column. The scaling was defined with targets *i*_6,5_ and *i*_6,8_. The location of these points used to define the coordinate system is not arbitrary and were selected as areas of maximum precision.

### 3.2. Tests for Accuracy Assessment

To study the metric potential of the measuring equipment, three tests were conducted to assess the photogrammetric precision, repeatability of results, and accuracy in the determination of coordinates. The first test was conducted only as a photogrammetric survey. In this test, photogrammetric precision was conceptually related to distances between the incident rays and the calculated position of the target. Formally, the precision values were measured in units of one standard deviation, based on the post-processing covariance matrix of the 3D object points. The photogrammetric precision includes systematic and random errors. Among the systematic errors are those derived from the limitations in modelling of the cameras and the divergence of the central projection as a result of the depth of field. Also included in the random errors are inaccuracies in the detection of targets as a result of limitations in terms of image resolution.

One of the fundamental limitations of the non-metric cameras is geometric instability or inability to maintain a constant internal geometry in the camera over time. The impact of this phenomenon was seen in the test of two situations: (1) using a set of parameters for internal camera geometry obtained just before the test without switching the camera on / off, and (2) using a set of internal parameters of the camera geometry obtained two months prior to the test in which there had been intense use of the cameras, including off / on cycling, and extension / retraction of the zoom.

In the second test of repetitiveness, there were 113 photogrammetric surveys with a total of 452 images. In all of the surveys, the test conditions were kept constant (position of the cameras, lighting, internal geometry parameters of the cameras, etc.). For each of the 113 photogrammetric surveys, 121 points were marked on the images, the external orientation of each camera was calculated (including bundle adjustment) and the coordinate system was defined. The repeatability in this test was analyzed through the standard deviation of the 113 coordinates of each point. The results show the overall repeatability of the method including all of the random errors of the phase of marking targets, those random errors introduced during the process of defining the coordinate system, and also that of scaling. All 113 photogrammetric surveys conducted in this study include the same systematic errors, so deviations in the coordinates are due solely to random errors.

**Figure 4.**
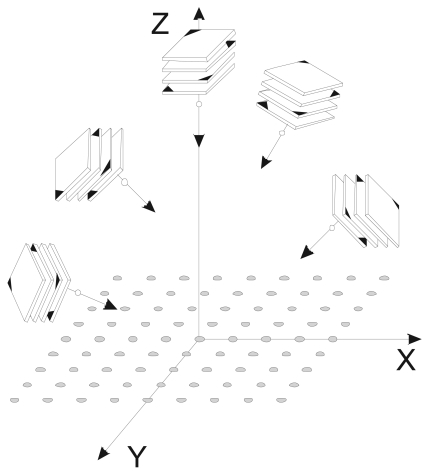
Network configuration of the photogrammetric survey to obtain “true” coordinates (third test for accuracy).

In the third test for accuracy evaluation, the coordinates obtained in the second test were compared with “true” coordinates obtained by a particularly robust photogrammetric survey. It consists in an improved configuration of images (Figure 4) which provides better ray intersections, higher redundancy and improved use of the image format [19]. In this survey, only the camera A was used, since it presents the lowest decentering distortion, the lowest marking residuals and the maximum photogrammetric precision (Table 3). Twenty images were taken from five stations by rotating the camera to 0°, 90°, 180°, and 270° in each station, with the intent to cover the entire test field in the central part of the sensor. With this configuration, the coordinates of 121 points were obtained with photogrammetric precision better than 0.022 mm in all directions of space and at any point of the field test.

Because the mean coordinates of the second test were used for comparison with “true” coordinates, this test is an assessment only of systematic errors resulting from the combined use of the four cameras in the system. This test of accuracy does not take into consideration matters related to the validity of standards used to scale the model.

## 4. Results and Discussion

### 4.1. First Test: Photogrammetric Precision

**Figure 5.**
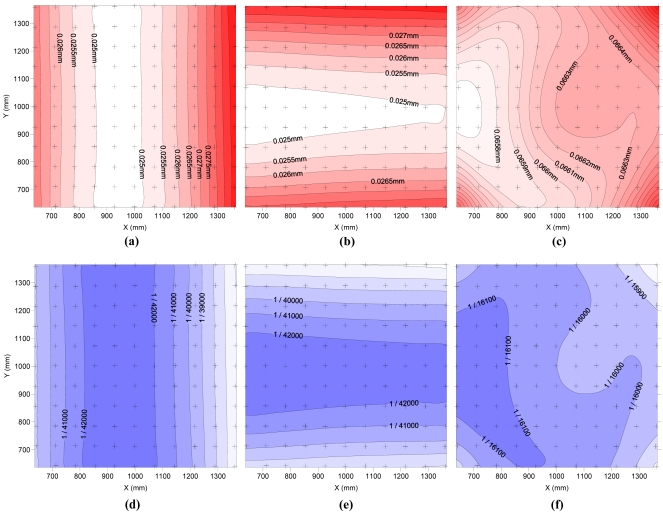
Spatial distribution of standard deviation (mm) obtained in the field test in the directions of X (a), Y (b) and Z (c). In (d), (e) and (f) standard deviation in the direction of X, Y and Z, respectively, is represented in relative units.

Figure 5 shows the photogrammetric precision achieved by the measuring equipment in the test field according to main directions X, Y and Z. The upper graphs represent the value of a standard deviation in millimetres for a normal distribution. The lower graphs also show standard deviations but in units relative to the maximum size of the field test (1,061 mm). In all graphs, the measurement points are indicated by black crosses.

As shown in Figure 5 the area of minor errors in the X direction is a central strip perpendicular to X where the standard deviations are smaller than 0.025 mm. Also the area of minor errors in the Y direction is a central strip perpendicular to Y where the standard deviations are smaller than 0.025 mm. In these areas of high precision targets are located closest to the cameras, besides the rays from the cameras converge on these areas with the same angle. It is also noted that the deviations grow as bands parallel to these areas to reach values of less than 0.030 mm. Standard deviations in the direction of Z take values in the entire test field of 0.065-0.066 mm except in the targets at the corner where values reached values slightly greater than 0.068 mm. Assuming normal distribution maximum deviations expected for a 95% probability are 0.049 mm as X, Y and 0.133 mm as Z. For a probability of 99.9% maximum deviations are expected 0.070 mm as X, Y and 0.190 mm as Z.

Figure 5c shows that the area of better precision in Z is on the left. The reason for this location may be related with residual systematic error in the modelling of cameras B and D, which were placed on the X-axe and present the highest decentering distortion and the highest residual markings (Table 3). In any case, the differences between the deviations in the rest of the test field are less than 0.001 mm. As can be seen in the graphs, the ratios of standard deviation and the size of the object are better than 1/35,000 for the X and Y directions and better than 1/15,000 for Z.

**Figure 6.**
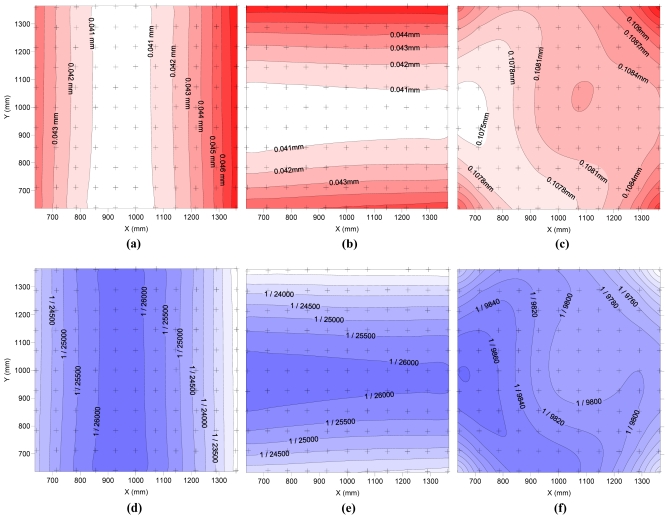
Spatial distribution of standard deviation (mm) obtained in the field test in the direction of X (a), Y (b) and Z (c) using old sets of modelling parameters for the cameras. In (d), (e) and (f) standard deviation in the direction of X, Y and Z, respectively, is represented in relative units.

Figure 6 shows the precision obtained with the same pictures but using old sets of modelling parameters of the cameras.

Comparison of Figures 5 and 6 shows that as areas of best and worst precision are maintained, this also maintains the ratio of precision between Z and X, Y. However, the highest standard deviations grow from 0.030 mm to 0.048 mm in the directions X and Y, and from 0.066 mm to 0.110 mm in the Z direction. These values represent precisions of over 1/9,500 for any direction. Assuming a normal distribution, maximum deviations expected for a 95% probability are 0.079 mm in X, Y and 0.148 mm in Z. For a probability of 99.9%, the maximum deviations are 0.148 mm for X, Y and 0.340 mm for Z. Although the use of an old set of parameters involves a significant worsening of the errors expected, the remaining precision can justify no need for modelling of the cameras with each use of the 3D measurement system, depending on the needs of the particular problem.

### 4.2. Second Test: Repeatability

**Figure 7.**
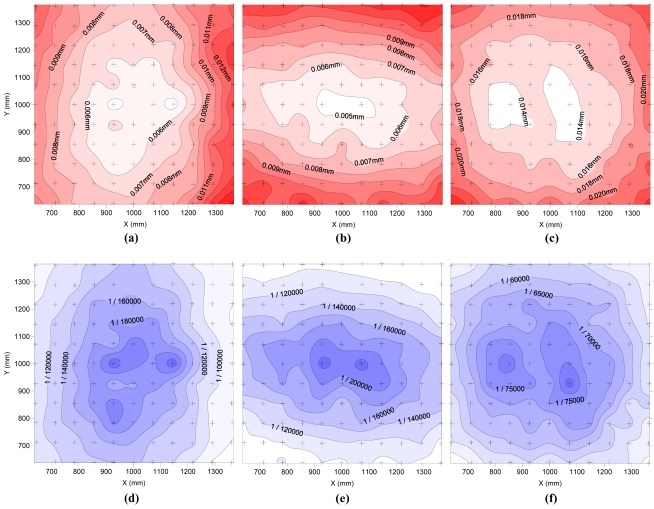
Spatial distribution of standard deviation (mm) obtained for the X (a), Y (b) and Z (c) components of the 113 calculated coordinates. In (d), (e) and (f) standard deviation of X, Y and Z components, respectively, is represented in relative units.

Figure 7 represents the standard deviations of the components X, Y and Z of the 113 calculated coordinates from each of the 121 points.

As in the first test of photogrammetric precision, there are areas of greater precision in X and Y. In this case, the areas are not as clearly defined as a strip, do not cross the entire test field, and are more intense in the centre. The area of greater accuracy in the direction of Z is at the centre of the test field. The values of standard deviation are below 0.013 mm for all points in the X and Y directions, and below 0.026 mm for all points in the direction of Z axis. Moreover, in a large area of a test field, standard deviations are lower than 0.008-0.010 mm in the X and Y directions, and 0.016 mm in the Z direction. As shown in the graphs below, this represents a relative precision better than 1/80,000 in the X and Y directions, and better than 1/40,000 in the Z direction. Deviations in the repeatability test are 3-5× lower than those obtained in previous testing of photogrammetric precision. The reason is for this is that in the repeatability test, systematic errors are equal in the 113 surveys and therefore do not cause variability in the results. Hence, the deviations obtained are caused only by random errors.

### 4.3. Third test: Accuracy

**Figure 8.**
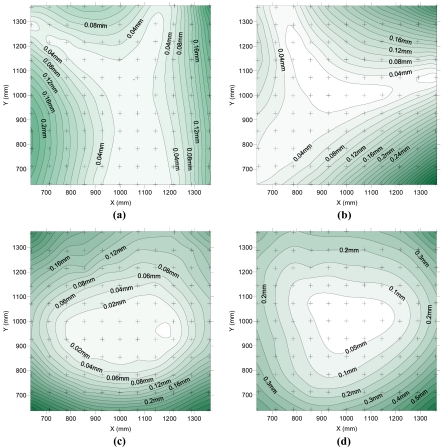
Spatial distribution of differences (mm) between the measurements and the “true” coordinates according to X (a), Y (b), Z (c) and the total vector length (d).

Figure 8 shows the difference, in absolute value and in millimetres, between the coordinates measured with the measurement system and the “true” coordinates in X, Y, Z. It also represents the total error vector as the quadratic sum of the components X, Y and Z. On this occasion, errors have not been represented in relative units because of differences very close to zero in the central area.

As shown in Figure 8, the maximum errors obtained are 0.240 mm in X and Y, while in Z, the errors can be up to 0.320 mm. These values are similar to those obtained considering a 99.9% probability in the photogrammetric precision test using the old set of modelling parameters (Figure 6). These values, in units relatives to the maximum size of the object, are 1/4,400 and 1/3,300, respectively. In a wide area in the middle of the test field, the errors obtained are much lower. It is estimated that over 50% of the test field area has a total error (quadratic sum of the three components) less than 0.200 mm, or in relative units, translate to accuracies better than 1/5,000.

As seen in Figures 5, 6, 7 and 8 the points placed in the centre show lower systematic and random errors and therefore more accurate determination of coordinates. These points are approximately equidistant to all cameras and hence the projected radiuses of targets in the image are similar in all the images. Peripheral points are close to one or two cameras but far from the others therefore the same target has different sizes of projected radius in the images. On the other hand, points located in the centre of the field test have a minimum lateral offset to optical axis, but it grows towards the periphery. The image radius of projected targets and the lateral offset to optical axis are two variables that affect the eccentricity that occurs as a result of the central projection of circular targets [19]. The eccentricities of the points in the centre of the field test are smaller and more uniform than eccentricities of the peripheral points. The lower accuracy in the marking of peripheral targets results in greater distance between convergent rays and lower precision and accuracy.

## 5. Summary and Conclusions

In this paper, we have presented a medium-accuracy optical measuring equipment system based on four low-cost consumer digital cameras. The cameras are synchronised to allow measurement of moving bodies (e.g., living beings). Tests were conducted to assess the metric potential of this equipment, separating systematic errors from random errors. It was shown that errors resulting from measurement of the coordinates are essentially systematic, and are derived from limitations in geometric modelling of the cameras and from eccentricity in the projected circular targets. Since the errors in determining coordinates are essentially systematic, the standard deviations in crossing of rays or marking residuals are not appropriate variables to describe the metric potential of the measuring equipment. A realistic description of the metric potential should use a confidence interval close to 100%. Finally, we have demonstrated that, with this equipment based in a low-cost multiple-camera and the use of circular targets, the 3D coordinates of any point common to all four cameras can be determined with an error less than 1/3,000 of the maximum size of the object, ie. sub-millimetre accuracy for an object with a size of one meter.

## Figures and Tables

**Photograph 1.**
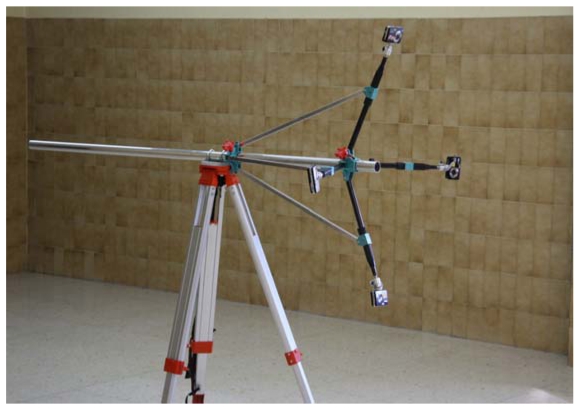
View of the 3D measurement system.

**Photograph 2.**
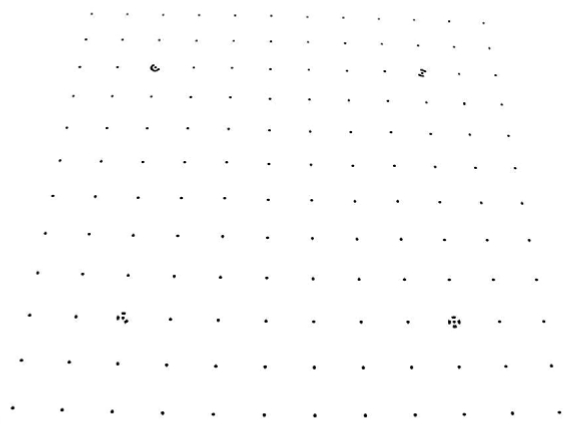
Plane point field used during calibration of the cameras.

**Photograph 3.**
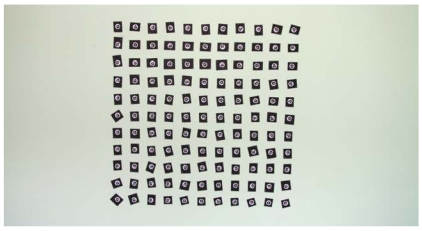
Field tests used to evaluate the potential of metric measurement equipment.

**Table 2.** Technical characteristics of cameras used in the measurement system (from http://www.dpreview.com/ and http://www.pentax.co.jp/).

**Feature**	**Pentax Optio A40**
Effective pixels	4,000 × 3,000
Image ratio w:h	4:3
Sensor size	1/1.7 inch, 7.60 × 5.70 mm, 0.43 cm^2^
Pixel density	28 MP/cm^2^
Pixel size	1.9 μm × 1.9 μm
Sensor type	CCD
Lens	7 elements in 5 groups (2 dual-sided aspherical elements, 1 single-sided aspherical element)
Focal Length	7.90 mm - 23.7 mm
Sensitivity	ISO 50-1600
Aperture	F2.8-F5.4
Shutter speed	4 s-1/2,000 s
File Formats	JPEG (EXIF 2.2)

**Table 3.** Interior orientation parameters and quality variables (image coverage, global point-marking residuals and global point precisions) obtained as results from modelling of the cameras.

	**Camera A**	**Camera B**	**Camera C**	**Camera D**
*C* (mm)	8.0547±2.1E-4	8.1895±3.2E-4	8.0913±3.4E-4	8.1239±3.7E-4
*x*′_0_ (mm)	-0.0588±1.8E-4	-0.1669±3.0E-4	-0.0622±2.5E-4	-0.1188±3.5E-4
*y*′_0_ (mm)	-0.0534±1.8E-4	-0.1797±2.9E-4	-0.0519±2.5E-4	-0.0785±3.4E-4
*k*_1_	3.15E-3±4.1E-6	2.89E-3±5.4E-6	3.10E-3±7.5E-6	2.97E-3±6.7E-6
*k*_2_	-3.00E-5±2.3E-7	-2.04E-5±3.1E-7	-2.81E-5±4.6E-7	-2.25E-5±3.7E-7
*B*_1_	7.18E-05±7.3E-7	3.50E-04±1.2E-6	-1.31E-04±9.5E-7	2.77E-04±1.3E-6
*B*_2_	-3.62E-04±6.7E-7	-3.27E-04±1.10E-6	-1.99E-05±9.3E-7	-5.31E-04±1.3E-6
*C*_1_	0.000035±3.4E-5	0.000139±5.3E-5	-0.000035±4.6E-5	0.000130±6.6E-5
Image coverage (%)	79	81	74	82
Overall RMS (pixels)	0.088	0.123	0.114	0.157
Overall RMS vector length (mm)	0.026	0.039	0.035	0.047

**Table 4.** Location of the centre of projection of cameras and orientation of the optical axes during the tests. ω, φ and κ are the angles between the optical axes and the object coordinate system. FOVh and FOVv are the horizontal and vertical angle of view of each camera.

**Camera**	**X** **(mm)**	**Y** **(mm)**	**Z** **(mm)**	**ω** **(°)**	**φ** **(°)**	**κ** **(°)**	**FOVh** **(°)**	**FOVv** **(°)**
A	970.92	1,459.85	1,177.79	-19.95	-0.48	0.71	49.8	38.4
B	516.09	957.27	1,192.54	0.57	-19.49	90.71	49.1	37.8
C	965.24	530.37	1,199.19	20.39	-3.10	179.08	49.7	38.3
D	1,436.67	978.59	1,201.20	2.19	18.46	-89.91	49.4	38.0
